# GPR43 activation-mediated lipotoxicity contributes to podocyte injury in diabetic nephropathy by modulating the ERK/EGR1 pathway

**DOI:** 10.7150/ijbs.64665

**Published:** 2022-01-01

**Authors:** Jian Lu, Pei Pei Chen, Jia Xiu Zhang, Xue Qi Li, Gui Hua Wang, Ben Yin Yuan, Si Jia Huang, Xiao Qi Liu, Ting Ting Jiang, Meng Ying Wang, Wen Tao Liu, Xiong Zhong Ruan, Bi Cheng Liu, Kun Ling Ma

**Affiliations:** 1 Institute of Nephrology, Zhongda Hospital, School of Medicine, Southeast University, Nanjing, 210009, China.; 2 John Moorhead Research Laboratory, Department of Renal Medicine, University College London (UCL) Medical School, Royal Free Campus, London, NW3 2PF, UK.

**Keywords:** diabetic nephropathy, podocytes, lipotoxicity, GPR43, autophagy, low-density lipoprotein receptor

## Abstract

**Background:** G-protein-coupled receptor 43 (GPR43) is a posttranscriptional regulator involved in cholesterol metabolism. This study aimed to investigate the possible roles of GPR43 activation in podocyte lipotoxicity in diabetic nephropathy (DN) and explore the potential mechanisms.

**Methods:** The experiments were conducted by using diabetic GPR43-knockout mice and a podocyte cell culture model. Lipid deposition and free cholesterol levels in kidney tissues were measured by BODIPY staining and quantitative cholesterol assays, respectively. The protein expression of GPR43, LC3II, p62, beclin1, low-density lipoprotein receptor (LDLR) and early growth response protein 1 (EGR1) in kidney tissues and podocytes was measured by real-time PCR, immunofluorescent staining and Western blotting.

**Results:** There were increased LDL cholesterol levels in plasma and cholesterol accumulation in the kidneys of diabetic mice. However, GPR43 gene knockout inhibited these changes. An* in vitro* study further demonstrated that acetate treatment induced cholesterol accumulation in high glucose-stimulated podocytes, which was correlated with increased cholesterol uptake mediated by LDLR and reduced cholesterol autophagic degradation, as characterized by the inhibition of LC3 maturation, p62 degradation and autophagosome formation. Gene knockdown or pharmacological inhibition of GPR43 prevented these effects on podocytes. Furthermore, GPR43 activation increased extracellular regulated protein kinases 1/2 (ERK1/2) activity and EGR1 expression in podocytes, which resulted in an increase in cholesterol influx and autophagy inhibition. In contrast, after GPR43 deletion, these changes in podocytes were improved, as shown by the *in vivo* and *in vitro* results.

**Conclusion:** GPR43 activation-mediated lipotoxicity contributes to podocyte injury in DN by modulating the ERK/EGR1 pathway.

## Introduction

The loss of glomerular filtration barrier (GFB) integrity and related albuminuria are features of diabetic nephropathy (DN). The GFB is composed of fenestrated endothelial cells, glomerular basement membrane and podocytes 1. Podocytopenia is an independent predictor of DN progression. However, the underlying mechanisms of podocyte injury in DN are not fully understood. Lipids and lipid-modulating proteins are key determinants of podocyte function. The podocyte slit diaphragm, which is assembled in lipid rafts, plays a critical role in the maintenance of GFB integrity 2. While excessive cholesterol accumulated in podocytes may cause lipotoxicity, such as endoplasmic reticulum stress, lysosomal impairment, and mitochondrial function disruption, which then further worsens metabolic disorders and the development of DN 3, 4. Clinical studies and experimental models of DN have shown that cholesterol accumulation in kidneys correlates with the development of glomerulosclerosis 5, 6. However, the cause of excessive cholesterol accumulation and the potential mechanisms of cholesterol trafficking disruption in podocytes under diabetic conditions are still unclear.

Short-chain fatty acid (SCFA) receptors belong to the superfamily of G protein-coupled receptors (GPRs). Among them, GPR43 mainly acts on acetate and propionic acid. GPR43 is highly expressed in immune cells 7, adipose cells 8 and islet cells 9, thus regulating inflammatory responses, lipogenesis and insulin secretion. In the kidney, GPR43 has been reported to be expressed in mesangial cells 10, distal renal tubules and collecting tubules 11. However, there have been few studies on the expression and metabolism of GPR43 in podocytes. Previous studies revealed that GPR43 activation promotes white 12 and brown 13 adipocyte differentiation and inhibits lipolysis. Moreover, GPR43 was involved in lipid accumulation in 1,3-dichloro-2-propanol (1,3-DCP) treated HepG2 cells 14. Compared with high-fat diet (HFD)-fed mice, HFD-fed GPR43-knockout (GPR43-KO) mice had lower body fat mass, lower plasma triglyceride and cholesterol levels, and less scattered lipids in brown adipose tissues 15. These results suggest that GPR43 may participate in lipid metabolic processes.

Autophagy is an evolutionarily conserved subcellular process, including the formation of autophagosomes and subsequent fusion with lysosomes 16. Lipophagy refers to the specific degradation of lipids by autophagy 16-18. The concept of lipophagy was first described in liver diseases, in which the destruction of gene of macroautophagy leads to the accumulation of lipid droplets 19. Lipophagy now appears in many cell types such as the pancreas and adipose tissues 20, 21. Cholesterol is stored in lipid droplets in the form of cholesterol ester (CE) and then transported to lysosomes, where CE is hydrolyzed by lysosomal acid lipase and free cholesterol is released. Notably, impaired autophagic flux and lysosomal function can cause excessive lipid accumulation, severe glomerular oxidative stress and inflammation 23. Recently, acetate-mediated GPR43 activation was shown to attenuate nucleotide-binding oligomerization domain (NOD)-like protein 3 (NLRP3) inflammasome activation by promoting autophagy 24. The link between autophagy and podocyte function has been demonstrated in both *in vitro* and *in vivo* 25, 26. Despite there are direct evidences of autophagy and GPR43 to date, little is known about the role of GPR43 in the regulation of autophagy and cholesterol homeostasis in podocytes.

Therefore, using a GPR43-knockout mouse model and a podocyte cell culture model, this study aimed to evaluate the potential lipotoxic effect of GPR43 activation on podocyte injury in DN and explore potential mechanisms.

## Methods

### Reagents and antibodies

The reagents of sodium acetate (Sigma-Aldrich) and PD0325901 (Selleck) were purchased. The summarized primary antibodies were used: GPR43 (Sigma-Aldrich), WT-1(Santa Cruz), extracellular regulated kinase (ERK) 1/2 and phosphorylated ERK1/2 (Cell Signaling Technology), early growth response protein 1 (EGR1) and LC3 (Proteintech), p62 and LDLR (Abcam), and secondary antibodies against goat anti-rabbit IgG (H+L) (Alexa Fluor 488) and goat anti-mouse IgG (H+L) (Alexa Fluor 594) (Proteintech) were used.

### Experimental mouse model

Heterozygous GPR43-KO mice were obtained from GemPharmatech Co., Ltd. (China). Genotyping was performed by polymerase chain reaction (PCR) analysis of genomic DNA preparations. Diabetes was induced via daily intraperitoneal injections of streptozotocin (STZ) (Sigma-Aldrich) (50 mg/kg) for 5 consecutive days. Blood glucose was measured 2 weeks post-injection. Mice with blood glucose levels greater than 16.7 mmol/L were considered diabetic. For the normal controls, animals were intraperitoneally injected with 0.1 mol/L sodium citrate buffer. The mice were then divided into four groups: control group (Ctrl), GPR43 knockout group (GPR43 KO), diabetes group (DM), and GPR43 knockout diabetes group (GPR43 KO + DM). Body weights were recorded, and urine samples were collected during the 12-week experimental period after diabetes induction. All procedures involving animals were performed in accordance with the Guide for the Care and Treatment of Laboratory Animals and was approved by the Institutional Animal Care and Use Committee at School of Medicine, Southeast University, China.

### Quantitative analysis of intracellular free cholesterol in the kidneys

Briefly, kidney tissue samples were exposed to a solution of anhydrous ethanol to isolate lipids. After the samples were homogenized with an ultrasound instrument and centrifuged at 10,000 ×g for 20 minutes, the supernatant was carefully collected. The cholesterol level in the supernatant was measured by using a free cholesterol kit according to an optimized method (Solarbio). The protein level was determined by using the Bradford assay with bovine serum albumin as a standard. The concentrations of free cholesterol in samples were calculated with a standard curve and normalized against the total cellular protein level.

### Immunofluorescent staining

Immunofluorescent staining was performed on formalin-fixed, paraffin-embedded kidney sections and cultured cells with diluted antibodies against WT-1 (1:50), LDLR (1:100), p62 (1:100), LC3 (1:200), and EGR1 (1:100). The immune complexes were detected by fluorescently labeled secondary antibodies. 4', 6-Diamidino-2-phenylindole (DAPI) (Beyotime) was used to stain the nuclei. The slides were visualized on a Zeiss LSM 700 confocal microscope (Carl Zeiss).

### Transmission electron microscopy (TEM)

After the treatment by 1% osmium tetroxide, podocyte samples were dehydrated with a graded series of acetone, and then embedded and polymerized. Ultrathin sections (50-70 nm) were prepared, stained with uranyl acetate and lead citrate, and observed by TEM (Hitachi).

### Cell culture and gene transfection in podocytes

Immortalized mouse podocytes were cultured as previously described 27. Briefly, podocytes were grown under permissive conditions at 33 °C in medium containing interferon-γ (IFN-γ), thermally shifted to 37 °C and differentiated for 10-14 days. Podocytes were treated with the ERK pathway inhibitor PD0325901 (5 μmol/L).

To specifically silence GPR43 and EGR1 mRNA expression, podocytes were grown to 60%-80% confluence and then transfected with a small interfering RNA (siRNA) duplex (GenePharma) specific for mouse GPR43, EGR1 or control siRNA by Lipofectamine 2000 reagent (Thermo Fisher Scientific) according to the manufacturer's instructions. The sequences of the siRNAs were as following: mouse GPR43, 5'-GGAUCUGAUCAAAGGCAUCTT-3'; mouse EGR1, 5'-AAAGGTGGTTTCCAGGTTCC-3'; and nonspecific scrambled siRNA, 5'-CCUACGCCACCAAUUUCGU-3'.

### Quantitative real-time PCR

Total RNA was extracted from podocytes or isolated renal cortex tissues using TRIzol reagent (Invitrogen). Reverse transcription was performed using HiScript III RT SuperMix (Vazyme) according to the manufacturer's instructions. Quantitative real-time PCR was performed using a 7300 AB real-time PCR machine (Applied Biosystems, USA) with ChamQ Universal SYBR qPCR Master Mix (Vazyme). The ΔΔCT of genes was normalized to the housekeeping mouse gene β-actin.

### Western blotting

Renal cortex tissues and podocytes were lysed in ice-cold RIPA buffer. Equal amounts of protein were separated by 10% sodium dodecyl sulfate-polyacrylamide gel electrophoresis (SDS-PAGE) and then transferred onto a polyvinylidene fluoride (PVDF) membrane. After being treated with blocking solution, the membranes were incubated with primary antibodies overnight at 4 °C, followed by incubation with peroxidase-conjugated anti-mouse or anti-rabbit IgG secondary antibodies for one hour. All target proteins were visualized by an enhanced chemiluminescence substrate. Densitometry was measured using ImageJ software (National Institutes of Health).

### BODIPY staining

Lipids in cells or sections were stained with 4,4-difluoro-1,3,5,7,8-pentamethyl-4-bora-3a,4a-diaza-s-indacene (BODIPY) (Thermo Fisher Scientific) for 30 minutes, followed by processing for immunofluorescence as described previously 28. Staining was checked by an Olympus confocal microscope (Olympus).

### LysoTracker Red and BODIPY costaining

The lysosomes in living cells were stained with 75 nmol/L LysoTracker Red (Beyotime) for 60 minutes at 37 °C according to the manufacturer's protocol, and podocytes were then fixed and stained with BODIPY for 30 minutes. The nuclei were visualized by DAPI staining.

### Statistical analysis

All data are presented as the mean values with standard deviation (SD). Statistical analysis was performed using GraphPad Prism 6 software. The results were analyzed using unpaired two-tail Student t-tests and one-way ANOVA. When one-way ANOVA showed statistical significance, the results were compared using a 2-tailed Student's t test after Tukey's correction for multiple comparisons. A *P* value less than 0.05 was considered statistically significant.

## Results

### GPR43 deficiency ameliorated cholesterol accumulation in podocytes

**Figure 1:**
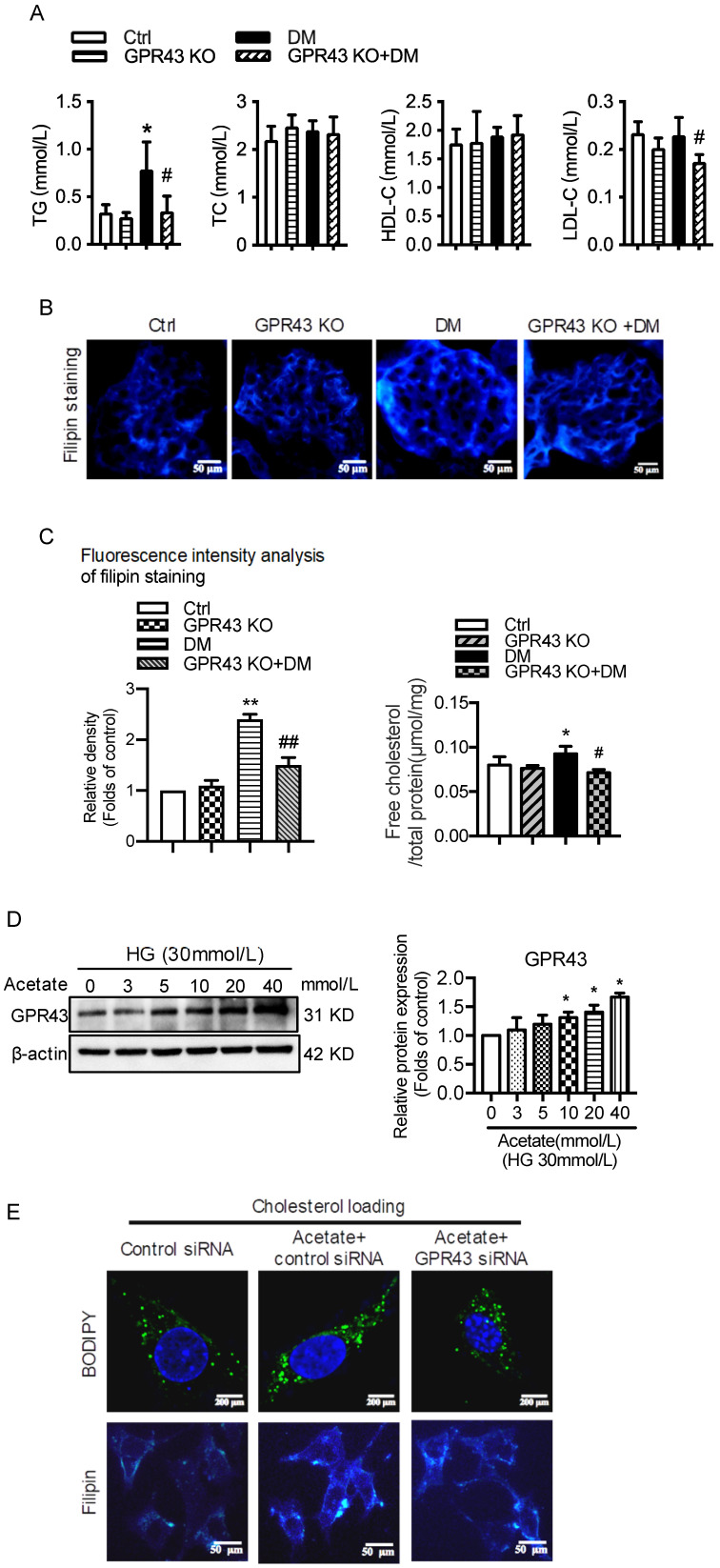

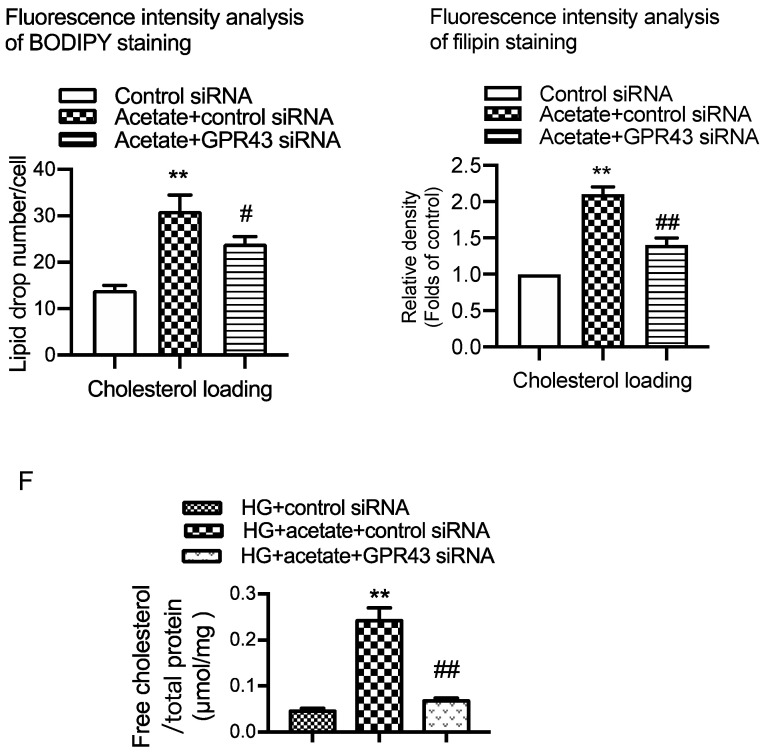
** GPR43 deficiency ameliorated cholesterol accumulation in podocytes in diabetic mice.** The mice were divided into four groups: the control group (Ctrl, n=6), GPR43-KO group (n=6), diabetes group (DM, n=6), and GPR43-KO diabetes mice (GPR43-KO+DM, n=7). The mice were treated for 12 weeks. **A.** The serum lipid profiles of mice were examined by clinical biochemical assays. The results are expressed as the mean ± SD. ^*^P < 0.05 *vs.* Ctrl, *^#^P*<0.05 *vs.* DM. TG, triglyceride; TC, total cholesterol; HDL-C, high-density lipoprotein cholesterol; LDL-C, low-density lipoprotein cholesterol. **B.** Cholesterol accumulation in the glomeruli of mice was examined by filipin staining. The fluorescence intensity analysis of filipin staining was presented. Original magnification × 400, scale bars, 50 µm (mean ± SD, ^**^P < 0.01 *vs.* Ctrl, *^##^P* < 0.01 *vs.* DM). **C.** Free cholesterol levels in the kidneys of mice were examined by quantitative free cholesterol assays (mean ± SD, ^*^P < 0.05 *vs.* Ctrl, *^#^P* < 0.05 *vs.* DM). **D.** The protein expression of GPR43 in podocytes was examined by Western blotting. Podocytes were treated with different concentrations of acetate in the presence of high glucose (HG) for 24 hours. The histogram shows the means ± SD of the densitometric scans of GPR43 protein bands following normalization to β-actin. ^*^P< 0.05 *vs.* HG. **E.** Podocytes were loaded with 30 µg/mL cholesterol in the presence or absence of acetate, GPR43 siRNA, or control siRNA (vehicle) for 24 hours and then examined by BODIPY staining (Green, BODIPY; blue, DAPI) and filipin staining. The fluorescence intensity analysis of BODIPY staining and filipin staining was presented. The images were captured by confocal microscopy (scale bar, 200 µm and 50 µm) (mean ± SD, ^**^P < 0.01 *vs.* control siRNA,^ #^*P* < 0.05, ^##^*P* < 0.01 *vs.* acetate + control siRNA). **F.** Cholesterol accumulation in podocytes was examined by quantitative free cholesterol assays. Podocytes were treated with or without acetate, GPR43 siRNA, or control siRNA (vehicle) for 24 hours in the presence of high glucose conditions (HG, 30 mmol/L). The concentration of free cholesterol was normalized to the total protein in cells (mean ± SD, ^**^P < 0.01 *vs.* HG + control siRNA, ^##^*P* < 0.01 *vs.* HG + acetate + control siRNA).

Plasma lipid profile analysis showed that GPR43 KO lowered the plasma levels of triglyceride (TG) and LDL-cholesterol (LDL-C) in diabetic mice, although there was no difference in the plasma levels of total cholesterol (TC) and high-density lipoprotein-cholesterol (HDL-C) among the four groups (Figure 1A). Filipin staining and free cholesterol quantitative assays were conducted to evaluate cholesterol deposition. The results showed that there was significantly increased cholesterol accumulation in the kidneys of diabetic mice. However, these effects were inhibited by GPR43 KO (Figure 1B and 1C). The results from the *in vitro* study demonstrated that acetate dose-dependently increased GPR43 protein expression under high glucose conditions (Figure 1D). Furthermore, acetate significantly induced lipid deposition in high glucose-stimulated podocytes. However, these effects were abrogated by GPR43 siRNA treatment, as demonstrated by BODIPY staining, filipin staining and free cholesterol quantitative assays (Figure 1E and 1F). These results suggest that GPR43 activation might be involved in lipotoxicity-mediated podocyte injuries in DN through the disruption of cholesterol homeostasis.

### GPR43 activation-mediated lipotoxicity in podocytes in DN could result from the upregulation of the LDLR pathway

**Figure 2:**
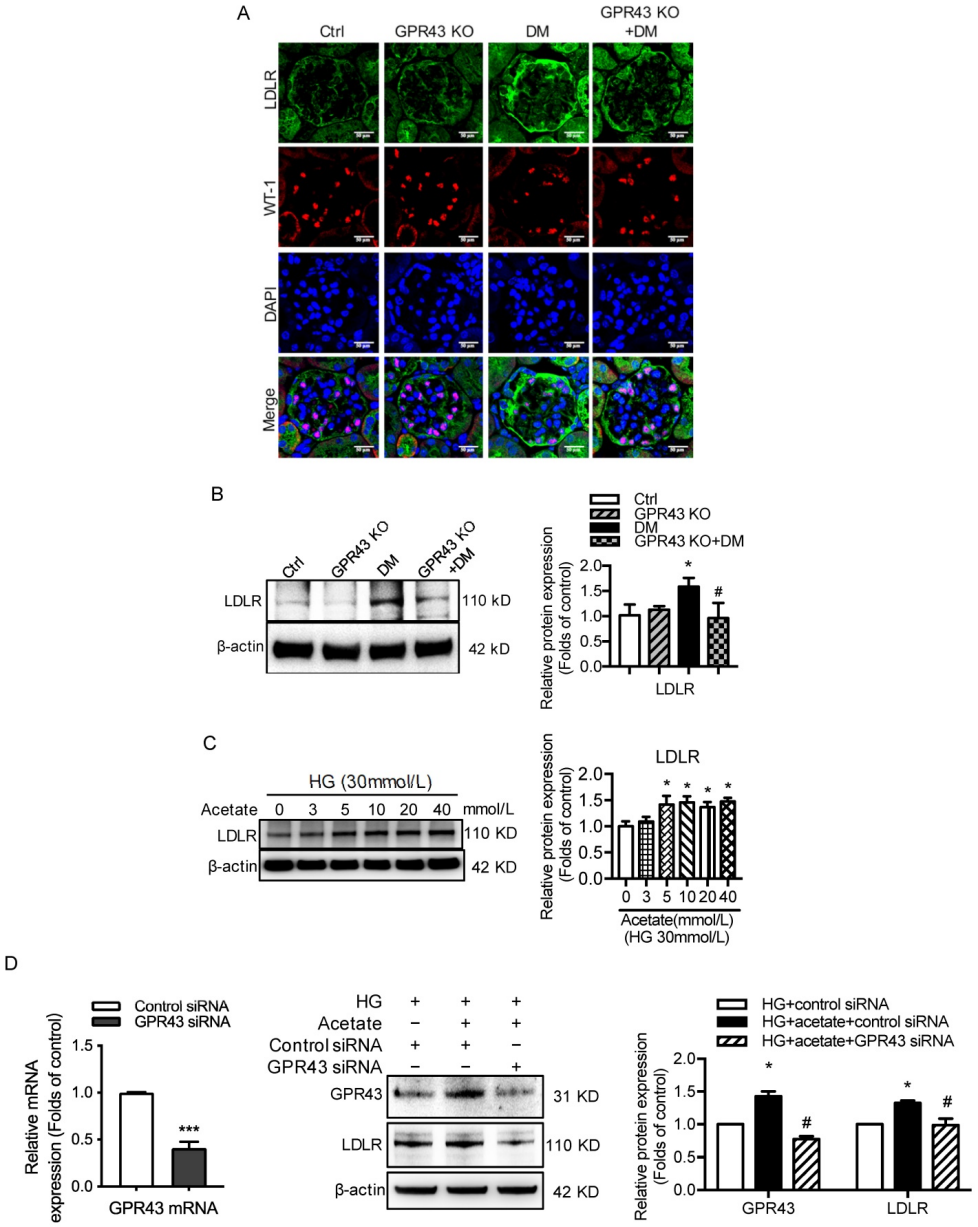
** GPR43 activation induced cholesterol accumulation in podocytes by increasing LDLR-mediated cholesterol uptake. A.** The protein expression of LDLR in podocytes in kidneys was examined by immunofluorescent staining. The mice were divided into four groups: the control group (Ctrl, n=6), GPR43-KO group (n=6), diabetes group (DM, n=6), and GPR43-KO diabetes mice (GPR43-KO+DM, n=7). The mice were treated for 12 weeks. The fluorescent signals of LDLR (green), WT-1 (red), and nuclei (blue) were measured by confocal microscopy (original magnification × 400, scale bars, 50 µm). LDLR: low-density lipoprotein receptor; WT-1, Wilm's tumor protein-1, a specific biomarker of podocytes. **B.** The protein expression of LDLR in kidneys was examined by Western blotting. The mice were divided into four groups: the control group (Ctrl, n=6), GPR43-KO group (n=6), diabetes group (DM, n=6), and GPR43-KO diabetes mice (GPR43-KO+DM, n=7). The mice were treated for 12 weeks. The histogram shows the means ± SD of the densitometric scans of LDLR protein bands following normalization to β-actin. ^*^P < 0.05 *vs.* Ctrl, *^#^P* < 0.05 *vs.* DM. **C.** The protein expression of LDLR in podocytes was examined by Western blotting. Podocytes were treated with different concentrations of acetate in the presence of HG for 24 hours. The histogram shows the means ± SD of the densitometric scans of LDLR protein bands following normalization to β-actin. ^*^P < 0.05 *vs.* HG. **D.** The mRNA expression of GPR43 was examined by real-time PCR. The protein expression of GPR43 and LDLR in podocytes was examined by Western blotting. Podocytes were treated with or without acetate, GPR43 siRNA, or control siRNA (vehicle) for 24 hours in the presence of HG conditions. The histogram shows the means ± SD of the densitometric scans of GPR43 and LDLR protein bands following normalization to β-actin. ^*^P < 0.05 *vs.* HG + control siRNA; ^#^*P* < 0.05 *vs.* HG + acetate + control siRNA.

To explore potential mechanisms of lipotoxicity in podocytes, we then examined the effects of GPR43 activation on the LDLR pathway in podocytes in DN. Immunofluorescent staining demonstrated that the protein expression of LDLR in the podocytes of diabetic mice. However, GPR43 KO abrogated the increase in LDLR expression in the podocytes of diabetic mice (Figure 2A). This effect was further confirmed by the Western blotting results (Figure 2B). The *in vitro* study showed that acetate dose-dependently increased the protein expression of LDLR in podocytes under high glucose conditions (Figure 2C). Acetate-mediated increase in LDLR protein expression in podocytes was inhibited by GPR43 siRNA, which correlated with a reduction in GPR43 protein expression (Figure 2D). These *in vivo* and *in vitro* findings suggest that the GPR43 activation-mediated upregulation of LDLR may induce lipotoxicity in podocytes in DN through an increase in cholesterol uptake.

### GPR43 activation inhibited the autophagy-lysosome pathway in podocytes in DN

**Figure 3:**
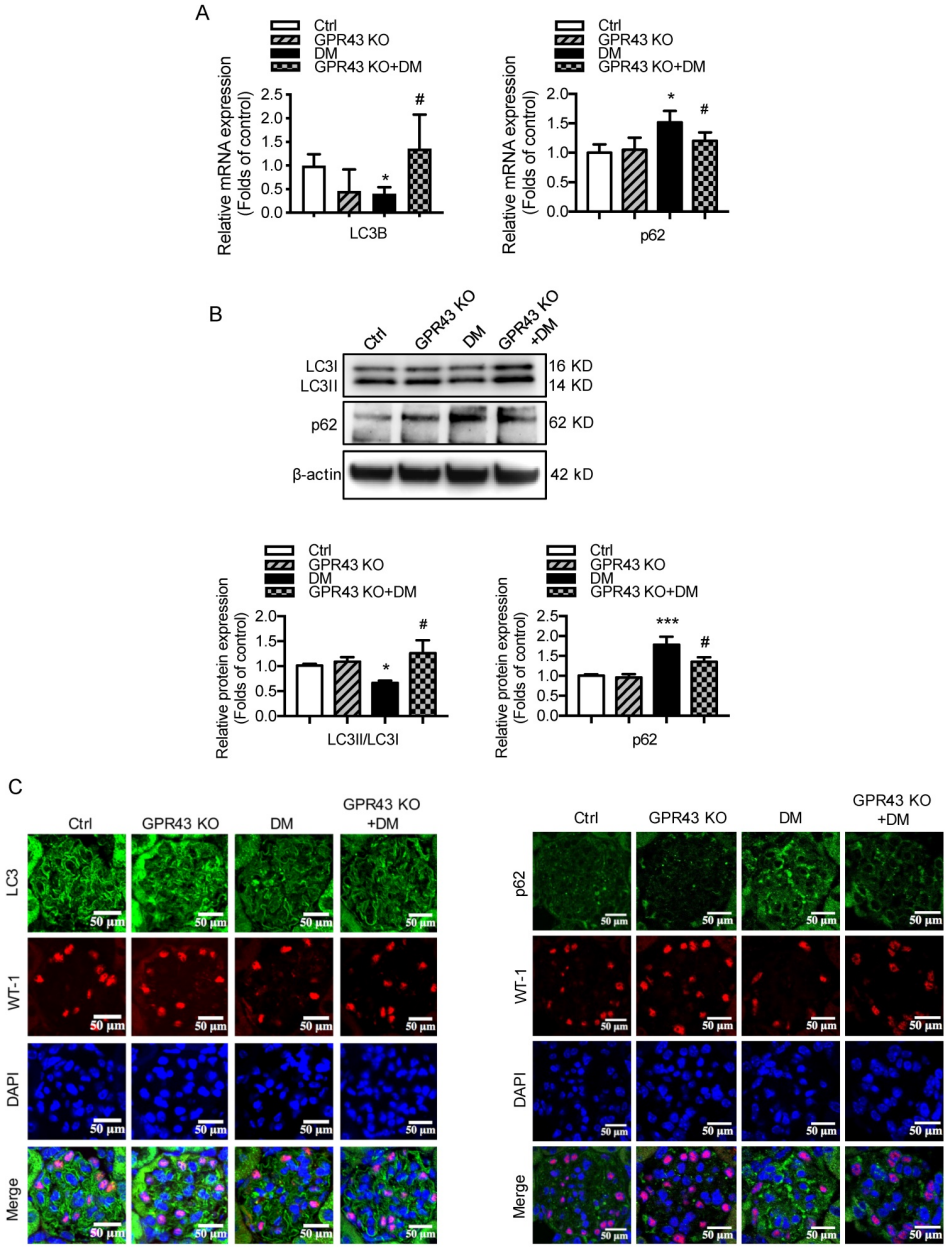

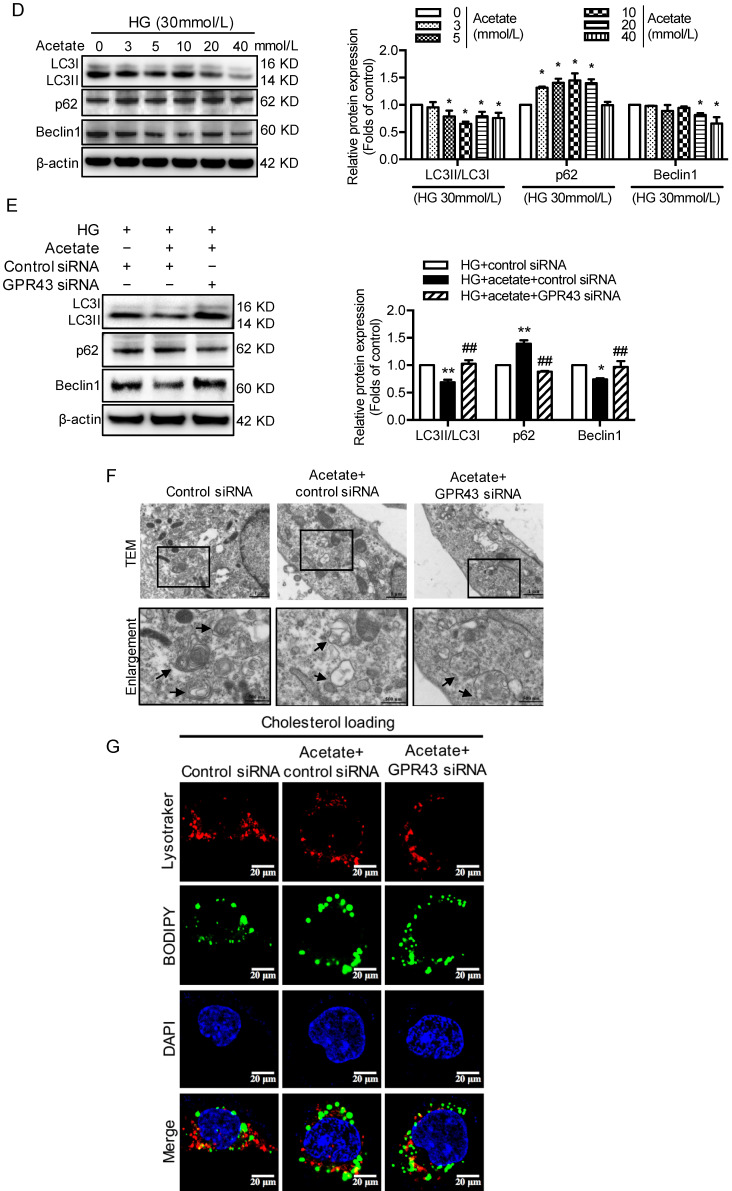
** GPR43 activation inhibited the autophagy-lysosome pathway in podocytes in DN. A.** The mRNA expression of LC3B and p62 in kidneys was examined by real-time PCR. The mice were divided into four groups: the control group (Ctrl, n=6), GPR43-KO group (n=6), diabetes group (DM, n=6), and GPR43-KO diabetes mice (GPR43-KO+DM, n=7). The mice were treated for 12 weeks. The mRNA expression of LC3B and p62 in the kidneys is expressed as the means ± SD. ^*^P < 0.05 *vs*. Ctrl, ^#^*P* < 0.05 *vs*. DM. **B.** The protein expression of LC3 and p62 in kidneys was examined by Western blotting. The mice were divided into four groups: the control group (Ctrl, n=6), GPR43-KO group (n=6), diabetes group (DM, n=6), and GPR43-KO diabetes mice (GPR43-KO+DM, n=7). The mice were treated for 12 weeks. The histogram shows the means ± SD of the densitometric scans of the LC3 and p62 protein bands following normalization to β-actin. ^*^P < 0.05, ^***^P < 0.001 *vs*. Ctrl, ^#^*P* < 0.05 *vs*. DM. **C.** The protein expression of LC3 and p62 in kidneys was examined by immunofluorescent staining. The mice were divided into four groups: the control group (Ctrl, n=6), GPR43-KO group (n=6), diabetes group (DM, n=6), and GPR43-KO diabetes mice (GPR43-KO+DM, n=7). The mice were treated for 12 weeks. The fluorescent signals of LC3 (green), P62 (green), WT-1 (red), and nuclei (blue) were examined by confocal microscopy. Original magnification × 400, scale bars, 50 µm. **C.** The protein expression of LC3, p62 and Beclin1 in podocytes was examined by Western blotting. Podocytes were treated with different concentrations of acetate in the presence of HG for 24 hours. The histogram shows the means ± SD of the densitometric scans of the LC3, p62, and Beclin1 protein bands following normalization to β-actin. ^*^P < 0.05 *vs.* HG. **D.** The protein expression of LC3, p62 and Beclin1 in podocytes was examined by Western blotting. Podocytes were treated with or without acetate, GPR43 siRNA, or control siRNA (vehicle) for 24 hours in the presence of high glucose conditions (HG, 30 mmol/L). The histogram shows the means ± SD of the densitometric scans of the LC3, p62, and Beclin1 protein bands following normalization to β-actin. ^*^P < 0.05, ^**^P < 0.01 *vs.* HG + control siRNA; ^##^*P* < 0.01 *vs.* HG + acetate + control siRNA. **E.** The ultramicrostructure of podocytes was analyzed by transmission electron microscopy (TEM). Podocytes were treated with or without acetate, GPR43 siRNA, or control siRNA (vehicle) for 24 hours. Representative TEM images showed that acetate-induced autophagy inhibition in podocytes was abrogated by GPR43 siRNA treatment. Typical autophagosome structures (as shown by black arrows) with partially degraded materials were identified in podocytes. **F.** Podocytes were loaded with 30 µg/mL cholesterol in the presence or absence of acetate, GPR43 siRNA, or control siRNA (vehicle) for 24 hours. Lysosome (LysoTracker, red) colocalization with lipids (BODIPY, green) was captured by confocal microscopy. Nuclei are shown in blue. Original magnification × 600, scale bars, 20 µm.

Hyperglycemia-induced autophagy insufficiency in podocytes is a hallmark of DN progression 29. The results showed that LC3B mRNA expression was decreased in the kidney tissues of diabetic mice compared to the controls. However, GPR43 KO abrogated the decrease in LC3B mRNA expression in diabetic mice (Figure 3A). Consistently, the mRNA expression of p62 was higher in diabetic mice than in controls, and this effect was reversed by GPR43 depletion (Figure 3A). Western blot analysis showed that the protein expression of LC3II and p62 in kidneys in the different groups was consistent with the mRNA level (Figure 3B). These results suggest that GPR43 deletion may induce the restoration of autophagy in diabetic mice. To further verify this finding, immunofluorescent staining was performed to examine the expression of LC3 and p62 in the podocytes of diabetic mice. There was increased LC3 staining in the kidneys of GPR43-KO diabetic mice compared to wild-type diabetic mice (Figure 3C). However, p62 expression in WT-1-positive podocytes from GPR43-KO diabetic mice was significantly lower than that in the controls (Figure 3C). Taken together, these data indicate that GPR43 activation may be involved in the inhibition of podocyte autophagy in diabetic kidneys, thereby resulting in reduced cholesterol degradation in podocytes.

The results from the *in vitro* study showed that acetate stimulation suppressed the LC3II/I ratio and Beclin1 protein expression (Figure 3D). Knockdown of GPR43 by siRNA restored the LC3II/I ratio and Beclin1 expression in podocytes (Figure 3E). p62 acts as an autophagy substrate. Autophagic flux deficiency is often accompanied by accumulation of the p62 protein. Our results showed that p62 protein expression was increased in acetate-stimulated podocytes (Figure 3D), whereas GPR43 siRNA treatment reversed the acetate-induced accumulation of p62 in podocytes (Figure 3E). Ultrastructural analysis by TEM showed that acetate inhibited the formation of autophagosomes, and GPR43 siRNA treatment restored the number of autophagosomes in podocytes (Figure 3F).

To further clarify whether autophagy is involved in GPR43-induced disruption of intracellular cholesterol homeostasis, we stained podocytes with the lysosomal marker LysoTracker Red together with BODIPY after 24 hours of treatment with acetate. The results showed that LysoTracker Red-BODIPY colocalization was significantly lower in acetate-treated podocytes than in controls. This effect was abrogated by GPR43 siRNA treatment (Figure 3G). This finding indicates that GPR43 may modulate the lysosome-related cholesterol degradation pathway. Thus, these* in vivo* and *in vitro* findings suggest that acetate-mediated GPR43 activation increases cholesterol accumulation in podocytes, which partly results from inhibition of the autophagy-lysosome pathway.

### GPR43 activation inhibited LDLR-mediated cholesterol uptake and autophagy-mediated cholesterol degradation in podocytes by modulating the EGR1 pathway

**Figure 4:**
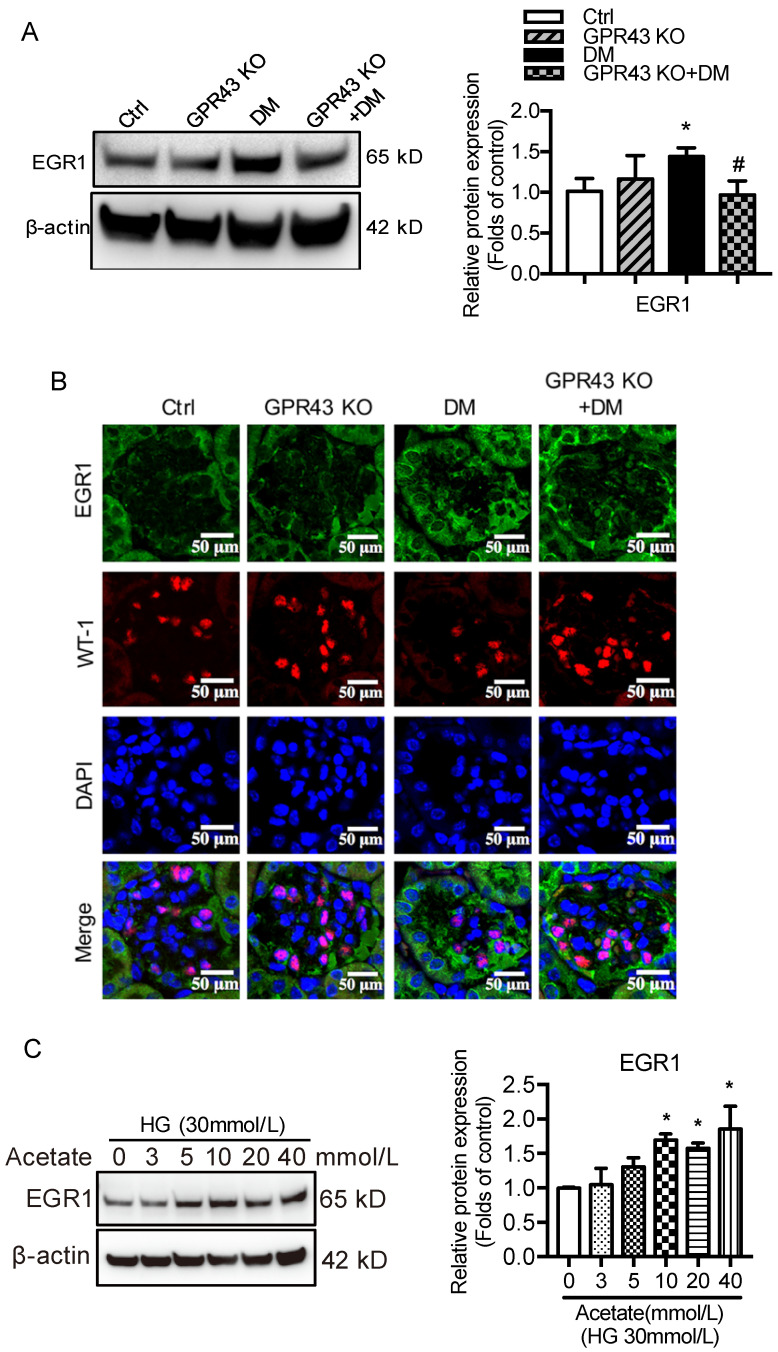

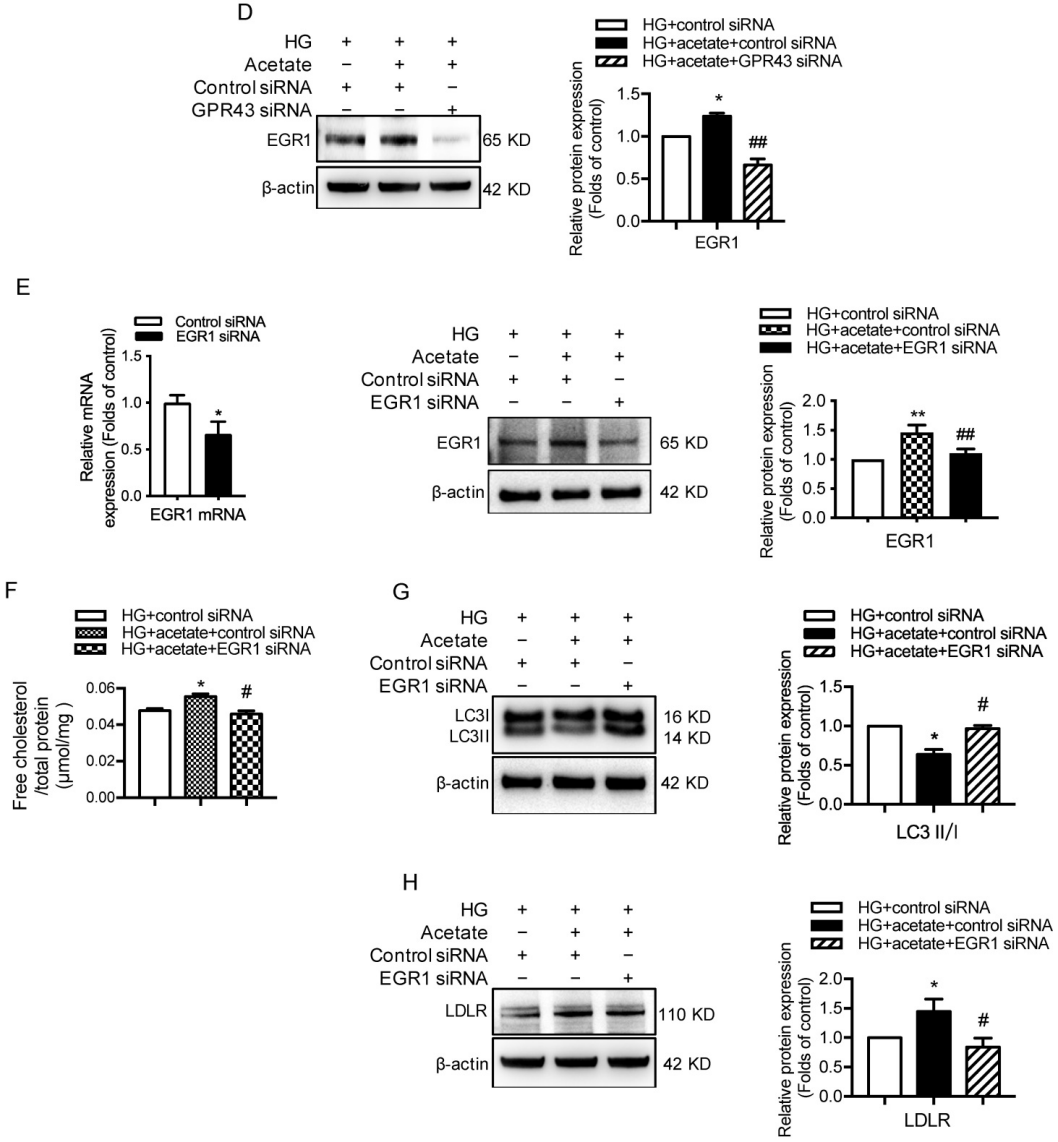
** GPR43 activation inhibited LDLR-mediated cholesterol uptake and autophagy-mediated cholesterol degradation in podocytes in DN by modulating the EGR1 pathway. A.** The protein expression of EGR1 in kidneys was examined by Western blotting. The mice were divided into four groups: the control group (Ctrl, n=6), GPR43-KO group (n=6), diabetes group (DM, n=6), and GPR43-KO diabetes mice (GPR43-KO+DM, n=7). The mice were treated for 12 weeks. The histogram shows the means ± SD of the densitometric scans of EGR1 protein bands following normalization to β-actin. ^*^P < 0.05 *vs*. Ctrl, ^#^*P* < 0.05 *vs*. DM. **B.** The protein expression of EGR1 in podocytes in kidneys was examined by immunofluorescent staining. The mice were divided into four groups: the control group (Ctrl, n=6), GPR43-KO group (n=6), diabetes group (DM, n=6), and GPR43-KO diabetes mice (GPR43-KO+DM, n=7). The mice were treated for 12 weeks. The fluorescent signals of EGR1 (green), WT-1 (red), and nuclei (blue) were examined by confocal microscopy (original magnification × 400, scale bars, 50 µm). EGR1: early growth response protein 1. **C.** The protein expression of EGR1 in podocytes was examined by Western blotting. Podocytes were treated with different concentrations of acetate in the presence of high glucose (HG, 30 mmol/L) for 24 hours. The histogram shows the means ± SD of the densitometric scans of EGR1 protein bands following normalization to β-actin. ^*^P < 0.05 *vs.* HG. **D.** The protein expression of EGR1 in podocytes was examined by Western blotting. Podocytes were treated with or without acetate, GPR43 siRNA, or control siRNA (vehicle) for 24 hours in the presence of high glucose conditions (HG, 30 mmol/L). The histogram shows the means ± SD of the densitometric scans of EGR1 protein bands following normalization to β-actin. ^*^P < 0.05 *vs*. HG + control siRNA; ^##^*P* < 0.01 *vs*. HG + acetate + control siRNA. **E.** The mRNA and protein expression of EGR1 in podocytes was respectively examined by real-time PCR and Western blotting. Podocytes were treated with or without acetate, EGR1 siRNA, or control siRNA (vehicle) for 24 hours in the presence of high glucose conditions (HG, 30 mmol/L). The histogram shows the means ± SD of the densitometric scans of EGR1 protein bands following normalization to β-actin. ^**^P < 0.01 *vs.* HG + control siRNA; ^##^*P* < 0.01 *vs.* HG + acetate + control siRNA. **F.** Cholesterol accumulation in podocytes was examined by quantitative free cholesterol assays. Podocytes were treated with or without acetate, EGR1 siRNA, or control siRNA (vehicle) for 24 hours in the presence of high glucose conditions (HG, 30 mmol/L). The concentration of free cholesterol was normalized to the total protein in cells (mean ± SD, ^*^P < 0.05 *vs.* HG + control siRNA, ^#^*P* < 0.05 *vs.* HG + acetate + control siRNA). **G-H.** The protein expression of LC3 (G) and LDLR (H) in podocytes was examined by Western blotting. Podocytes were treated with or without acetate, EGR1 siRNA, or control siRNA (vehicle) for 24 hours in the presence of high glucose conditions (HG, 30 mmol/L). The histogram shows the means ± SD of the densitometric scans of LC3 and LDLR protein bands following normalization to β-actin. ^*^P < 0.05 *vs.* HG + control siRNA; ^#^*P* < 0.05 *vs.* HG + acetate + control siRNA.

Next, we investigated potential signaling pathways that could mediate the GPR43 activation-induced upregulation of LDLR expression and autophagy inhibition. Previous studies have revealed that EGR1 functions as central transcriptional regulator of autophagy by modulating autophagic flux 30. The EGR1 also regulates LDLR transcription via the interaction with CCAAT/enhancer binding protein β (C/EBPβ) in HepG2 cells 31. Thus, we further explored whether the EGR1 mediated GPR43 activation-induced upregulation of LDLR expression and autophagy inhibition. From Western blotting and immunofluorescent staining, we found that the protein expression of EGR1 was significantly increased in the kidneys of diabetic mice. However, this effect was inhibited by GPR43 KO (Figure 4A and 4B). *In vitro* study further confirmed that acetate stimulation increased EGR1 protein expression in podocytes (Figure 4C), and this effect was abolished by treatment with GPR43 siRNA (Figure 4D). These results indicate that acetate-mediated GPR43 activation upregulates EGR1 expression. Therefore, we next examined whether the EGR1 pathway was involved in the modulation of cholesterol homeostasis. EGR1 siRNA treatment abolished the acetate-induced increase in EGR1 expression. The quantitative free cholesterol assay demonstrated that (Figure 4E) EGR1 knockdown auppressed the acetate-induced free cholesterol accumulation in podocytes (Figure 4F). Interestingly, there was decreased LC3II protein expression in podocytes in response to acetate stimulation, which was improved by EGR1 siRNA treatment (Figure 4G). In addition, EGR1 siRNA treatment suppressed acetate-mediated upregulation of LDLR protein expression in podocytes (Figure 4H). These results indicate that the EGR1 pathway may be involved in GPR43 activation-mediated cholesterol accumulation in podocytes in DN via the upregulation of the LDLR pathway and autophagy inhibition.

### GPR43 activation upregulated the expression of EGR1 in podocytes in DN by increasing ERK1/2 activation

**Figure 5:**
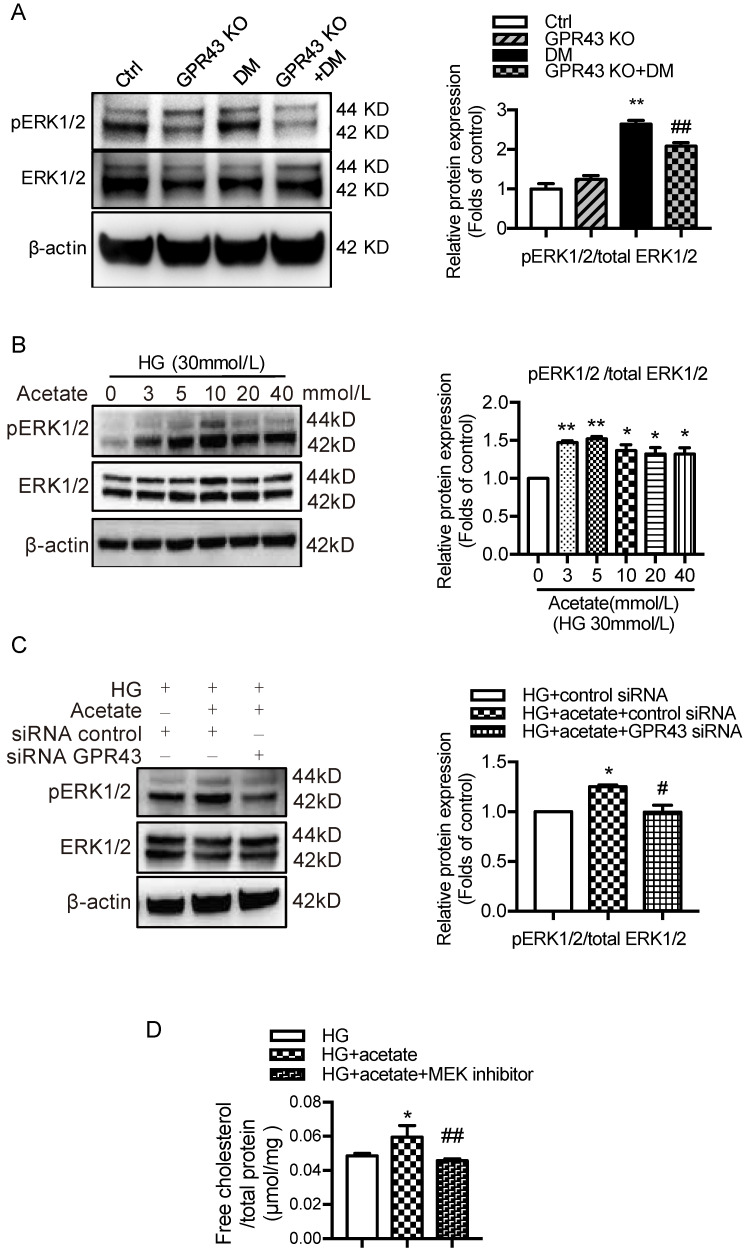

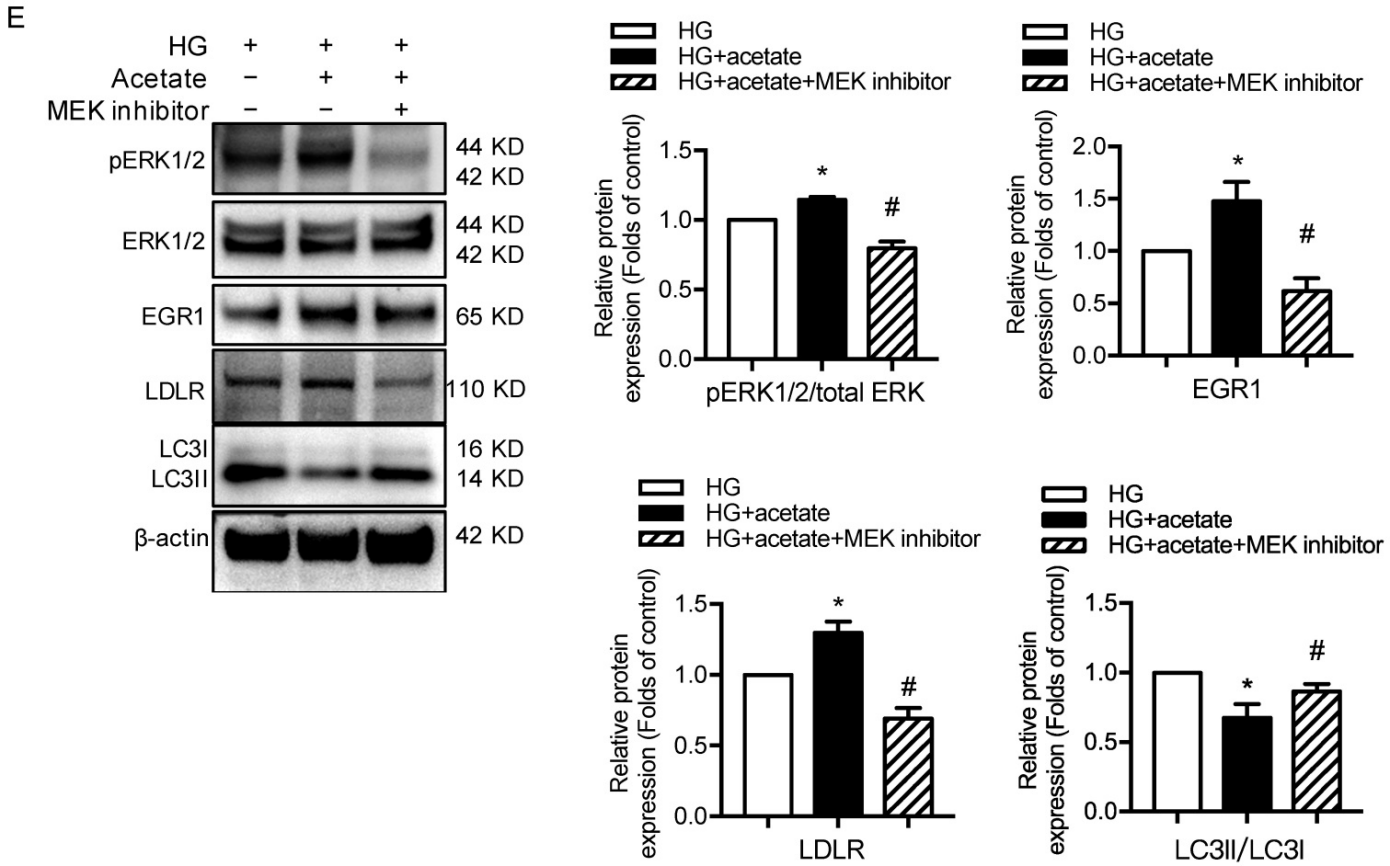
** GPR43 activation upregulated the expression and transcriptional activity of EGR1 in podocytes of DN by increasing the nuclear translocation of ERK1/2. A.** ERK1/2 protein phosphorylation in kidneys was examined by Western blotting. The mice were divided into four groups: the control group (Ctrl, n=6), GPR43-KO group (n=6), diabetes group (DM, n=6), and GPR43-KO diabetes mice (GPR43-KO+DM, n=7). The mice were treated for 12 weeks. The histogram shows the means ± SD of the densitometric scans of phosphorylated ERK1/2 protein bands following normalization to β-actin (^**^P < 0.01 *vs*. Ctrl, ^##^*P* < 0.01 *vs*. DM). **B.** ERK1/2 protein phosphorylation in podocytes was examined by Western blotting. Podocytes were treated with different concentrations of acetate in the presence of high glucose (HG, 30 mmol/L) for 24 hours. The histogram shows the means ± SD of the densitometric scans of ERK1/2 phosphorylated protein bands following normalization to β-actin. ^*^P < 0.05,^ **^P < 0.01 *vs*. HG. **C.** ERK1/2 protein phosphorylation in podocytes was examined by Western blotting. Podocytes were treated with or without acetate, GPR43 siRNA, or control siRNA (vehicle) for 24 hours in the presence of high glucose conditions (HG, 30 mmol/L). The histogram shows the means ± SD of the densitometric scans of ERK1/2 phosphorylated protein bands following normalization to β-actin. ^*^P < 0.05 *vs.* HG + control siRNA; ^#^*P* < 0.05 *vs.* HG + acetate + control siRNA. **D.** Cholesterol accumulation in podocytes was examined by quantitative free cholesterol assays. Podocytes were treated with or without acetate and a MEK inhibitor (PD0325901, 5 µmol/L) for 24 hours in the presence of high glucose conditions (HG, 30 mmol/L). The concentration of free cholesterol was normalized to the total protein in cells. (mean ± SD, ^*^P < 0.05 *vs.* HG; ^##^*P*<0.01 *vs.* HG + acetate). **E.** The protein expression of pERK1/2, EGR1, LDLR, and LC3 in podocytes was examined by Western blotting. Podocytes were treated with or without acetate and a MEK inhibitor (PD0325901, 5 µmol/L) for 24 hours in the presence of high glucose conditions (HG, 30 mmol/L). The histogram shows the means ± SD of the densitometric scans of the pERK1/2, EGR1, LDLR and LC3 protein bands following normalization to β-actin. ^*^P < 0.05 *vs.* HG; *^#^P* < 0.05 *vs.* HG + acetate.

A recent study reported that EGR1 expression was modulated by ERK signaling pathway 32. To explore potential mechanisms by which GPR43 activation mediates upregulation of EGR1, we examined the ERK pathway. The results showed that ERK1/2 phosphorylation was increased in diabetic kidneys, and this effect was abolished by GPR43 KO (Figure 5A). An *in vitro* study showed that acetate increased ERK1/2 phosphorylation in podocytes in a dose-dependent manner under high glucose conditions (Figure 5B), while GPR43 siRNA treatment effectively inhibited acetate-induced ERK1/2 phosphorylation (Figure 5C). Taken together, these findings suggest that GPR43 activation upregulates the activity of ERK1/2 in podocytes.

ERK1/2 signaling has been previously shown to regulate EGR1 activity in HepG2 cells via direct phosphorylation. Moreover, EGR1 modulates the transcriptional activation of the LDLR gene 31. Thus, we sought to determine whether GPR43 regulates the EGR1 signaling pathway in podocytes through ERK1/2, thereby regulating intracellular cholesterol homeostasis. The results showed that acetate treatment increased the transcriptional upregulation of EGR1 in podocytes, and this effect was inhibited by blocking the ERK1/2 pathway using the well-characterized MEK inhibitor PD0325901 (Figure 5E). This finding suggests that ERK1/2 activity is required for activation of the EGR1 pathway in podocytes upon acetate challenge. As predicted, the level of free cholesterol was significantly increased in acetate-stimulated and cholesterol-loaded podocytes; however, this effect was inhibited by PD0325901 (Figure 5D). Further analysis showed that the increased cholesterol accumulation in podocytes correlated with the upregulation of LDLR protein expression induced by ERK/EGR1 pathway activation (Figure 5E). In addition, our results showed that ERK1/2 inhibition mediated by PD0325901 resulted in decreased LDLR expression and increased LC3II/I expression in podocytes (Figure 5E). This finding suggests that the ERK1/2 pathway may regulate cholesterol endocytosis and autophagy in podocytes. In summary, these findings indicate that GPR43 activation may increase LDLR-mediated cholesterol endocytosis and inhibit autophagy-dependent cholesterol degradation in podocytes via the modulation of the ERK/EGR1 pathway.

## Discussion

Our study demonstrated that GPR43 deficiency downregulated LDLR-mediated cellular cholesterol uptake and restored podocyte autophagic flux to facilitate CE degradation, which exerted a robust protective effect by reducing cholesterol loading in podocytes in DN. We further suggested that GPR43 promoted the ERK1/2 signaling cascade in podocytes, thereby increasing EGR1 and LDLR expression, and autophagy impairment. These findings highlight the key role of GPR43 in regulating the effect of autophagy on cholesterol metabolism.

Substantial evidence supports that GPR43 activation modulates systemic and tissue-specific energy metabolism, especially in insulin resistance and obesity 33. Studies have revealed that compared to HFD-fed mice, HFD-fed mice with GPR43 deletion had lower levels of plasma lipids and body fat mass and improved glucose control 15, 34. Additionally, GPR43 activation promoted adipogenesis and enhanced triglyceride hydrolysis and free fatty acid oxidation in adipose tissues, accompanied by body weight reduction 35. In contrast, inconsistent results have shown that GPR43-deficient mice are obese on a normal diet, and mice with GPR43 specifically overexpressed in adipose tissues remain lean even on a HFD. The GPR43 activation inhibits insulin signaling and fat accumulation in adipose tissues and promotes lipid and glucose catabolism in other tissues. In addition, a HFD induces an increase in serum acetate levels and the expression of GPR43 in islet β cells. Compared to normal HFD-fed mice, GPR43-deficient mice fed a HFD developed glucose intolerance with defects in insulin secretion. Moreover, GPR43-deficient mice fed a HFD exhibited reductions in β-cell mass and the expression of differentiation genes 8. These findings suggest that GPR43 acts as an effector of excessive dietary energy, thereby accelerating body energy utilization to maintain metabolic homoeostasis. Therefore, it has been suggested that the upregulation of GPR43 in diet-induced obesity occurs in a tissue-specific manner 36. Our previous study demonstrated that GPR43 activation induced by dysbiosis of the intestinal microbiota mediated tubulointerstitial injury by disrupting cholesterol homeostasis 37. Importantly, our current data demonstrated that GPR43 depletion alleviated lipid accumulation in glomerular podocytes in DN, which was accompanied by significant decreases in serum LDL cholesterol levels. Thus, GPR43 may represent a potential novel treatment target for diabetic podocyte injury.

Podocyte injury and loss are early pathological changes in the pathogenesis of DN. Increased intracellular cholesterol results in the accumulation of toxic glucose metabolites, exacerbating the development of DN. Physiologically, the cholesterol level in podocyte plays a specific role in maintaining podocyte structure and function. Intracellular cholesterol homeostasis is maintained through the coordinated regulation of cholesterol synthesis and intracellular trafficking 38. Circulating LDLs, which are the main carriers of cholesterol, are absorbed into lysosomes in an LDLR-dependent manner. Among cellular organelles, ATP-binding cassette subfamily A member 1 (ABCA1) enables cholesterol efflux to HDL acceptors. In the present study, we found that GPR43 activation promoted podocyte cholesterol overload by two novel mechanisms: the promotion of cholesterol uptake and impaired intracellular degeneration processes due to autophagy inhibition.

Autophagy is a process of degrading damaged organelles and misfolded proteins. Impairment of autophagy could be one of the main mechanisms of podocyte metabolic disorder in early DN. Previous studies have revealed that podocyte-specific deletion of ATG5 (encoding a protein that is important for phagocytic membrane elongation) leads to autophagy arrest, which results in proteinuria and increased susceptibility to glomerular diseases such as DN and focal segmental glomerulosclerosis 39, 40. Previous studies have shown that LDL is transferred to lysosomes through endosomes after LDL binds to LDLR in macrophages, while oxidized LDL (ox-LDL) is primarily taken up by scavenger receptors through endocytosis. CEs in LDL and ox-LDL can be hydrolyzed in lysosomes. Thus, impairments in lysosomal function and autophagy may lead to the excessive accumulation of CEs to form foam cells. Our study demonstrated for the first time that GPR43 activation inhibited autophagic flux in podocytes, which further blocked CE hydrolysis and facilitated excessive intracellular cholesterol accumulation. However, the knockdown of GPR43 restored autophagic flux. In addition, GPR43 activation decreased both the level of LC3II and the number of autophagosomes, suggesting that GPR43 activation impedes the formation of autophagosomes.

Autophagy is regulated by a variety of signaling pathways, including the mammalian target of rapamycin (mTOR) pathway and ERK1/2 pathway. The ERK1/2 pathway could induce the transformation of LC3I to LC3II and promote the production of Beclin1 41, both of which can induce autophagosome formation. It is well known that many ligand-bound GPRs activate ERK1/2 signaling and further regulate cell growth, division and differentiation. We further investigated the potential mechanisms of GPR43 activation on autophagy through ERK1/2 activation. Surprisingly, acetate stimulation induced ERK activation in podocytes, while treatment with GPR43 siRNA prevented ERK activation. Consistent with these data, the ERK1/2 pathway was activated in the kidneys of diabetic mice, while this stimulatory response of ERK1/2 was weakened in GPR43-deficient diabetic mice. Our findings suggest that ERK1/2 serves as an important downstream intercellular signaling molecule for the inhibition of autophagy by GPR43.

EGR1 is the target gene of ERK1/2 and was previously considered an additional transcription factor that upregulates the expression of cholesterol biosynthesis genes. EGR1 expression is upregulated by growth factors and hypoxia 42, 43, and has been shown to modulate the actin cytoskeleton and cell death 44, 45. Several studies have demonstrated that EGR1 upregulation induces glomerular mesangial cell proliferation 46 and accelerates renal interstitial fibrosis through activation of the TGF-β signaling pathway 47. EGR1 expression in podocytes was increased in mice with proteinuria, while the loss of EGR1 reduced proteinuria and glomerulosclerosis in mice 48. However, the function of EGR1 in DN is still unclear. In this study, we performed* in vivo* and *in vitro* experiments and demonstrated that GPR43 deficiency decreased EGR1 protein expression in podocytes under hyperglycemia. Furthermore, knockdown of EGR1 by siRNA or inhibiting ERK1/2 activity reduced LDLR protein expression but increased the expression of LC3II in podocytes. These results suggest that the GPR43/ERK/EGR1 pathway may be involved in the modulation of cholesterol homeostasis in podocytes in DN through LDLR-mediated cholesterol uptake and the inhibition of autophagy.

**Figure 6:**
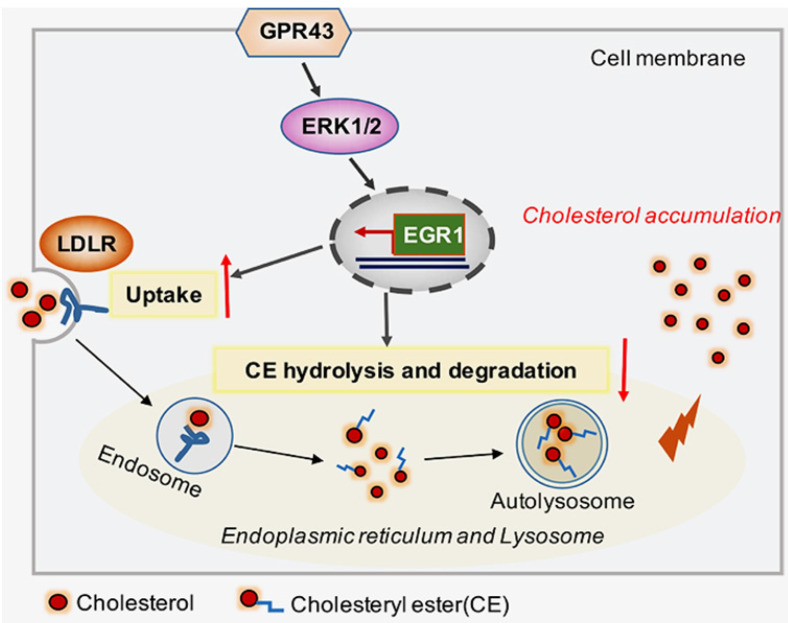
Schematic diagram showing that GPR43-mediated lipotoxicity contributed to podocyte injury in DN. Under high glucose conditions, acetate-mediated GPR43 activation stimulated cholesterol uptake in podocytes through the upregulation of LDLR and inhibited cholesterol hydrolysis and degradation by inhibiting autophagy. The potential mechanism was mainly mediated by modulation of the GPR43/ERK/EGR1 pathway. Moreover, EGR1 activation was a critical factor that governs both LDLR-mediated cholesterol uptake and autophagy-mediated cholesterol degradation, ultimately leading to cholesterol accumulation in podocytes and DN progression.

In conclusion, our study discovered that GPR43 activation increases cholesterol accumulation in podocytes and contributes to disease progression of DN. Here, we provided several lines of evidence that the GPR43/ERK/EGR1 axis regulates podocyte cholesterol metabolism and that GPR43 depletion protects against cholesterol accumulation in podocytes and glomerular injuries in diabetic mice (Figure 6). Therefore, antagonists targeting GPR43 are likely to be an effective approach to prevent podocyte injury and halt DN progression.
